# Special Issue: Advances in Dental Bio-Nanomaterials

**DOI:** 10.3390/ma15062098

**Published:** 2022-03-12

**Authors:** Satoshi Komasa, Joji Okazaki

**Affiliations:** Department of Removable Prosthodontics and Occlusion, Osaka Dental University, 8-1 Kuzuha-hanazono-cho, Hirakata 573-1121, Osaka, Japan; joji@cc.osaka-dent.ac.jp

**Keywords:** dental materials, biomaterials, dental implants, in vitro analysis, in vivo analysis, antibacterial analysis

## Abstract

Recent advances in dental materials involving the development of various biomaterials have been reported. Accordingly, clinicians must incorporate the new dental materials in their practice to respond to the increasing needs of patients. Nanotechnology is defined as a science that deals with nanoscale materials. The use of nanomaterials is gaining popularity in the dental industry for processing and manipulating nanoscale substances in modern dentistry. In this special issue, we invited the submission of several research papers on the development of dental materials. In this general discussion, we briefly explain the relevant research reports with an aim that developments in this field will contribute toward the development of dental care in the future.

Nanotechnology is the science which deals with materials at the nanometer scale. Its application to medical treatment is called nanomedicine. Although certain related techniques have already been practically implemented, new diagnostic and therapeutic methods using nanoscale particles and devices are still being researched. Consequently, nanotechnology has been applied to dentistry (especially in implant treatment, which is at the center of dentistry). In recent years, implant treatment has emerged as an indispensable procedure in prosthetic treatment, and it is performed in various oral environments. Although early osseointegration is one such case, nanostructuring of the material surface aims to modify the initial behavior of the bone marrow cells. Moreover, implant treatment is impossible without bone grafting in several cases, in which minimally invasive and safe implant treatment can be performed if the bone formation is fulfilled using only a bone-filling material. Principally, nanomaterials are advantageous for early bone formation, and future research results may stipulate the development of dental materials with excellent properties. In addition to implant treatment, diagnostic and treatment methods using nanoscale devices have been applied to dentistry. Thus, in this special issue, we introduce a number of such methods to our readers, expecting that these methods will be implemented in future dental treatments.

Bone regeneration using mesenchymal stem cells pose several limitations. Nakano et al. investigated adipose-derived dedifferentiated fat (DFAT) cells as an alternative and evaluated their cell proliferation rate, osteoblast differentiation, and bone regeneration ability in combination with activated platelet-rich plasma (aPRP) [1]. Thereafter, they isolated rat DFATs and aPRP using ceiling culture and centrifugation, respectively, which revealed that the cell proliferation rate was significantly increased by the addition of aPRP. Additionally, alizarin red staining was positive 21 d after the initiation of induction, with significantly higher Runt-related transcription factor 2 (Runx2) and osteocalcin expression levels than those in the controls, wherein a 9 mm critical defect was almost reasonably closed (60.6%). Therefore, the combination of DFAT cells with aPRP may constitute an effective approach for bone regeneration.

As such, the transplantation of engineered three-dimensional (3D) bone graft substitutes form a viable approach for regenerating severe bone defects. For large bone defects, an appropriate 3D scaffold may be required to support and stimulate bone regeneration, even if a sufficient number of cells and cell cytokines are available. In this study, Adachi et al. evaluated the in-vivo performance of a nanogel tectonic 3D scaffold, specifically developed for bone tissue engineering, referred to as nanogel cross-linked porous-freeze-dry (NanoCliP-FD) gel [2]. Subsequently, the spectroscopic imaging of the bone tissue grown in vivo upon application of NanoCliP-FD gel enabled the evaluation of bone quality, and thus can be employed to assess the feasibility of NanoCliP-FD gel scaffolding as a therapeutic modality for bone diseases associated with large bone defects.

Bio-C Sealer is a recently developed high-plasticity, calcium-silicate-based, ready-to-use material. In context, X-ray diffraction (XRD), scanning electron microscopy (SEM), and Fourier-transform infrared (FTIR) analyses were employed on the hydrated calcium silicate produced in the presence of water molecules. In addition, FTIR spectroscopy revealed the formation of calcium hydroxide and polyethylene glycol, a dispersing agent. The 1:4 dilutions of Bio-C Sealer exhibited weaker cytotoxicity than Calcipex II in an in-vitro system using the V-79 cell line. After 90 d, the periradicular tissue response of beagle dog roots was histologically evaluated. An absence of periradicular inflammation was reported in 17 of the 18 roots assessed with the Bio-C Sealer, whereas mature vertical periodontal ligament fibers were observed in the apical root ends filled with the Bio-C Sealer. Based on these results and prior investigations, the Bio-C sealer can be recommended as an effective root-end filling material. These results are relevant for clinicians considering the application of Bio-C Sealer for the treatment of patients [3].

Early treatment of dental caries is essential for maintaining the quality of life. To date, the clinical diagnosis of dental caries has been based on the subjective assessment by each dentist. However, this visual method lacks objectivity. To improve diagnostic ability, highly sensitive quantitative methods have been developed to diagnose and prevent dental caries, which are gradually emerging as a mandatory item in modern dentistry. Herein, we build upon our previous findings in a new analysis of dental caries using Raman spectroscopy imaging and discuss the possibility of applying Raman photonic imaging in support of objective diagnostics in dentistry. These results imply that the Raman methodology and related diagnostic algorithms, as applied herein, could represent a breakthrough in dentistry. Raman spectroscopy could enable rapid and safe in-vivo assessments of enamel and dentin quality as well as provide a unique path for developing reliable procedures associated with caries risk prediction [4].

Freeze-drying, also known as lyophilization, is widely used for preparing porous biomaterials. Nevertheless, limited information is available regarding the effect of gas permeability on molds to obtain porous materials. Han et al. demonstrated that the various levels of gas permeability of molds remarkably altered the pore distribution of the prepared gelatin sponges and distinct bone formation at critical-sized bone defects of the rat calvaria [5]. A gelatin sponge prepared using the ST mold demonstrated a wider pore size and spatial distribution with larger average pore diameter (149.2 µm) compared to those prepared using STPL and STPLB. More specifically, sponges using ST demonstrated significantly poor bone formation and bone mineral density after three weeks. Thus, the results signify that the gas permeability of molds critically alters the pore size and spatial pore distribution of the prepared sponges during the freeze-drying process, which probably causes distinct bone formation.

In Matsumoto’s study [6], a quartz crystal microbalance (QCM) was used to measure the nanogram-level amount of protein and bone marrow cells adhered to the surfaces of titanium (Ti) surface in real time. Additionally, the effects of ultraviolet (UV) and atmospheric-pressure plasma treatments on the surface hydrophilicity of the implant surface were evaluated as well. Consequently, the surface treatment methods remarkably decreased the surface C content and increased the O content along with superhydrophilicity. Overall, the QCM measurement results demonstrated an enhancement in the adhesion of both adhesive proteins and bone marrow cells after surface treatment. Although both methods produced implants with appropriate osseointegration behavior and less reactive oxidative species, the samples treated with atmospheric-pressure plasma delivered the most suitable overall performance and are recommended for clinical use, which verified the effectivity of QCM as a method for analyzing the initial adhesion process of dental implants.

Comba et al., evaluated the effectiveness of chemical-based adhesive techniques for promoting immediate and aged bond strength between zirconia and luting cement [7]. ANOVA analysis unveiled that artificial aging significantly impacts the bond strength of Zr. Moreover, the Bonferroni test highlighted the considerable influence of adhesive treatment (Signum) on bond strength after thermocycling. Conclusively, the 10-MDP-based bonding systems displayed no improvement in the initial bond strength compared to the tribochemical treatment. Relatively, all of the chemical bonding techniques tested in this study were influenced by thermocycling.

Thereafter, Yamaguchi [8] prepared and evaluated a biologically active biphasic bone substitute to promote bone formation by inducing precipitation and growth of HA crystals on the surface of a bone substitute. Biphasic bone substitute granules were prepared by immersing HA granules in a supersaturated calcium phosphate solution prepared by mixing five medical infusion solutions, and subsequently the precipitate was analyzed and the biological activities of the biphasic HA granules were evaluated in vitro and in vivo. Consequently, the precipitated calcium phosphate crystals were identified as low-crystalline HA, which grew markedly as needle-shaped crystals on the surface of the HA granules and significantly promoted cell proliferation and bone differentiation. In animal experiments, biphasic HA granules exhibited significantly higher bone mineral density, new bone volume ratio and new bone area ratio, implying that biphasic hydroxyapatite is a useful bone substitute for bone augmentation in dental implant treatment.

Oral dysfunction caused by peri-implantitis and the shortened life of implants has emerged as a major concern. Self-care and removal of oral biofilms by professional mechanical tooth cleaning (PMTC) are indispensable for its prevention. Therefore, Sato devised a new blasting cleaning method using agar particles that does not adversely affect the implant surface [9]. The simulated stains were almost completely removed upon blasting them with white alumina (WA) abrasive grains and CaCO_3_ particles, and the surface roughness transformed into a satin-finished surface, which was initially considered to be caused by fine scratches and thereby increased the surface roughness. Overall, most of the simulated stains were removed from the sample surface blasted with glycine and agar particles. Conversely, the gloss of the sample surface was maintained after cleaning, and the surface roughness increased slightly.

Although treatments for enamel demineralization or acid erosion are available, they posed certain limitations in their application. Chen et al. [10] aimed to manufacture a device that could directly form a hydroxyapatite (HAp) film coating on the enamel with a chairside erbium-doped yttrium aluminum garnet (Er:YAG) laser using the pulsed laser deposition (PLD) method for repairing enamel defects [10]. Subsequently, SEM images demonstrated that the α-TCP powder converted into microparticles upon irradiation, and the XRD peaks revealed that the coatings were almost completely hydrolyzed into HAp within two days. Moreover, the micro-Vickers hardness indicated that the hardness initially lost by decalcification was almost entirely recovered by the coatings. Furthermore, the results suggested that the Er:YAG-PLD technique was advantageous for repairing enamel defects and holds great potential for future clinical applications.

It was shown that many new dental materials and evaluation devices have been developed. It is necessary for these to have a function that does not have a harmful effect on the living body and contributes to the maintenance and improvement of a healthy life. For this purpose, verification at the nano level is necessary, and many verifications are needed in the future.
